# Sex and *FOXP3* gene rs2232365 polymorphism may be associated with the clinical and pathological aspects of chronic viral diseases

**DOI:** 10.1186/s12865-020-00387-4

**Published:** 2020-11-19

**Authors:** Leonn Mendes Soares Pereira, Max Willy da Silva Madureira, Renata Bezerra Hermes de Castro, Isabella Nogueira Abreu, Simone Regina Souza da Silva Conde, Sâmia Demachki, Maisa Silva de Sousa, Maria Alice Freitas Queiroz, Andrea Nazaré M. Rangel da Silva, Sandra Souza Lima, Marluísa de Oliveira Guimarães Ishak, Ricardo Ishak, Antonio Carlos Rosário Vallinoto

**Affiliations:** 1 Virology Laboratory, Biological Sciences Institute, Federal University of Pará (Universidade Federal do Pará – UFPA), Belém, Brazil; 2 Hematology and Hemotherapy Center Foundation of the State of Pará (Fundação Centro de Hematologia e Hemoterapia do Estado do Pará), Belém, Brazil; 3 Medical School, Biological Sciences Institute, UFPA, Belém, Brazil; 4 Tropical Medicine Center, UFPA, Belém, Brazil

**Keywords:** FOXP3, Polymorphism, Sex, Chronic viral diseases

## Abstract

**Background:**

The forkhead box protein 3 (FOXP3) transcription factor is one of the main markers of immunological suppression in different pathological profiles, and the presence of polymorphic variants may alter the gene expression of this factor. Despite descriptions of an association between the presence of the rs2232365 polymorphism and chronic diseases, the role of the sex variant in this context has not yet been elucidated, as the *FOXP3* gene is located on the human sex chromosome X.

**Results:**

To contribute to this topic, 323 women and 373 men were enrolled in the study, of which 101 were diagnosed with chronic viral liver diseases (39 women and 62 men), 67 with HTLV-1 infection (44 women and 23 men), 230 with coronary artery disease (91 women and 139 men) and 298 healthy and uninfected blood donors (149 women and men). They were genotyped for the rs2232365 polymorphism. The rs2232365 polymorphism was associated with clinical and pathological aspects and biomarkers of viral infections only in men, with functional differences between different infections.

**Conclusions:**

A relationship is suggested between sex and *FOXP3* rs2232365 polymorphism, resulting in different biological repercussions.

## Background

The forkhead box protein 3 (FOXP3) transcription factor is the main marker of regulatory T-cell (Treg) activation, a subpopulation specialized in the suppression of immune responses and the maintenance of homeostatic tolerance in different microenvironments [1]. The impact of Treg frequency on the progression of chronic diseases is important because these cells mediate the inflammatory balance in an immunologically polarized environment [2].

*FOXP3* gene is located on the short arm of the human sex chromosome (X on XY model) (Xp11.23; 21 kb) and consists of 11 exons [3]. The relevance of single nucleotide polymorphisms (SNPs) located in the promoter region of the *FOXP3* gene has been investigated due to they are involved in the transcription activation, as well as in the interaction with regulatory elements of the gene expression, possibly reflecting on the level of expression of FOXP3 and, thus, in the activation of Treg cells [4, 5].

In this context, the rs2232365 polymorphism in the *FOXP3* gene is related to functional changes that predispose individuals to diseases due to the mutant allele (C) changing the binding site of transcription factors that interact with the region [6]. Although the knowledge about the association of this polymorphism with different diseases is based on the literature [7, 8], the relationship between genetic variants of *FOXP3* and sex, remain unclear. Thus, the present study evaluated the association of the rs2232365 polymorphism with clinical and pathological aspects and markers of progression of viral and non-viral chronic diseases between the sexes.

## Results

**Fig. 1:**
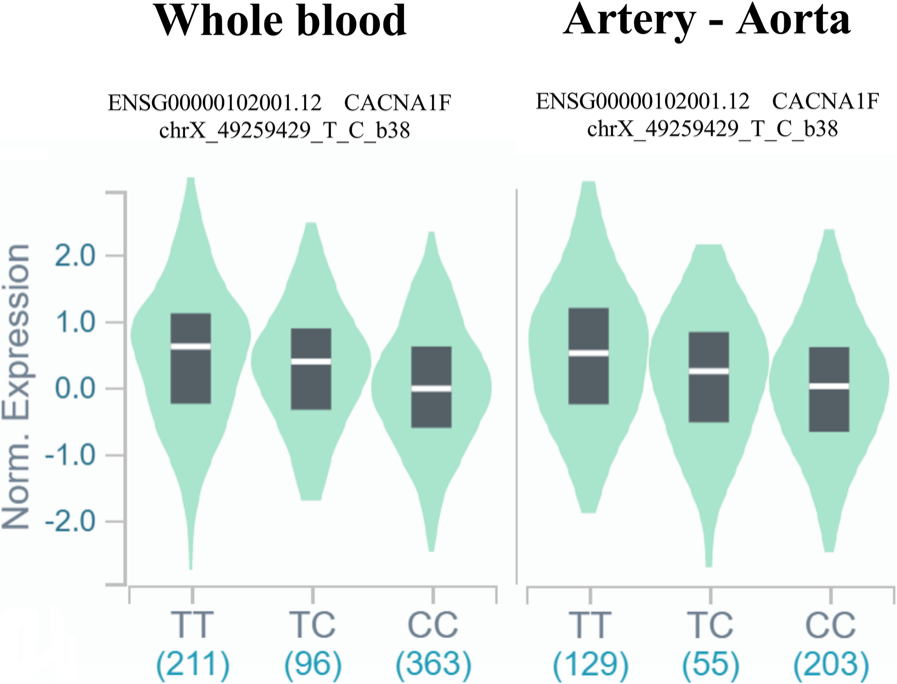
Biological implications of the rs2232365 polymorphism: Gene expression graph containing normalized *FOXP3* expression data by genotype of the rs2232365 polymorphism in whole blood and arterial tissue (GText portal). The numbers subscribed to the genotypes correspond to the total of samples collected

At first, we researched the biological meaning of the rs2232365 polymorphism in public databases of gene expression (GText portal - https://www.gtexportal.org/home/snp/rs2232365). The TT genotype has the highest level of expression of the *FOXP3* gene in different tissues investigated (Fig. 1). We collected expression data in whole blood and arterial tissue, the bank does not yet have liver tissue data.

In our results, the allele frequency of the rs2232365 polymorphism was similar between sexes, however statistical differences were observed between genders when assessing viral infections (Table 1). The polymorphism was in Hardy-Weinberg equilibrium (*p* > 0.05).

**Table 1:** Association of sex and allele frequency of the rs2232365 polymorphism in patients with chronic diseases, stratified according to the clinical-pathological profile

	Sex			Alleles (rs2232365) Female (%)			Alleles (rs2232365) Male (%)			Multiple logistic regression
	Female (323)	Male (373)	Statistic^**a**^	T	C	Statistic^**a**^	T	C	Statistic^**a**^	
**Group**				349 (54.03)	297 (45.98)	–	188 (50.40)	185 (49.60)	–	
CVLD	39 (12.07)	62 (16.62)	0.07	39 (50.00)	39 (50.00)	0.47	31 (50.00)	31 (50.00)	0.55	NS
HTLV-1	44 (13.62)	23 (06.16)	0.03 1.91 (1.10–3.33)	52 (59.09)	36 (40.91)	0.62	7 (33.33)	16 (66.67)	0.05 0.36 (0.14–0.92)	Sex*: 2.11 (1.18–3.75) T*: 0.43 (0.20–0.95)
CAD	91 (28.17)	139 (37.27)	0.02 1.53 (1.08–2.16)	93 (51.10)	89 (48.90)	0.41	68 (48.92)	71 (51.08)	0.35	Sex*: 1.44 (0.95–2.19)
CG	149 (46.13)	149 (39.95)		165 (55.40)	133 (44.63)		82 (54.31)	67 (45.70)		
**Inflammation (CVLD)**										
A0-A1	24 (61.54)	43 (69.35)	0.52	24 (50.00)	24 (50.00)	1.0	20 (57.90)	23 (53.49)	0.58	
A2-A3	15 (38.46)	19 (30.65)		15 (50.00)	15 (50.00)		11 (46.51)	8 (42.11)		
**Fibrosis (CVLD)**										
F0-F1	20 (51.28)	29 (46.77)	0.82	20 (50.00)	20 (50.00)	1.0	10 (34.48)	19 (65.52)	0.05 4.18 (1.13–15.4)	T*: 1.11 (0.43–2.90)
F2	8 (20.51)	17 (27.42)		8 (50.00)	8 (50.00)		10 (58.82)	7 (41.18)		
F3-F4	11 (28.21)	16 (25.81)		11 (50.00)	11 (50.00)		11 (68.75)	5 (31.25)		
**HTLV-1**										
Asymptomatic	27 (61.36)	13 (61.90)	1.0	32 (59.26)	22 (40.74)	0.86	4 (30.77)	9 (69.23)	0.87	
HAM/TSP	17 (38.64)	8 (38.10)		20 (58.82)	14 (41.18)		3 (37.50)	5 (62.50)		
**Risky infections (CAD)**										
*Chlamydia*+	78 (85.71)	121 (87.05)	0.84	80 (86.02)	74 (85.06)	1.0	60 (88.24)	61 (85.92)	0.80	
*Chlamydia*-	13 (14.29)	18 (12.95)		13 (13.98)	13 (14.94)		8 (11.76)	10 (14.08)		
**Species of** ***Chlamydia***										
*C. pneumoniae*	55 (70.51)	82 (67.77)	0.74	58 (70.73)	56 (66.67)	0.99	43 (71.67)	39 (63.93)	0.50	
*C. trachomatis*	3 (03.85)	3 (02.48)		2 (02.44)	2 (02.38)		2 (03.33)	1 (01.64)		
Coinfection	20 (25.64)	36 (29.75)		22 (26.83)	20 (23.81)		15 (25.00)	21 (34.43)		

*CVLD* Chronic Viral Liver Diseases

*CAD* Coronary Artery Disease

*CG* Control Group

^**a**^x^2^ test. Odds ratio (Confidence interval - 95%)

^*^0.05 ≥ *p* ≥ 0.01

^**^0.01 ≥ *p* ≥ 0.001

**Fig. 2:**
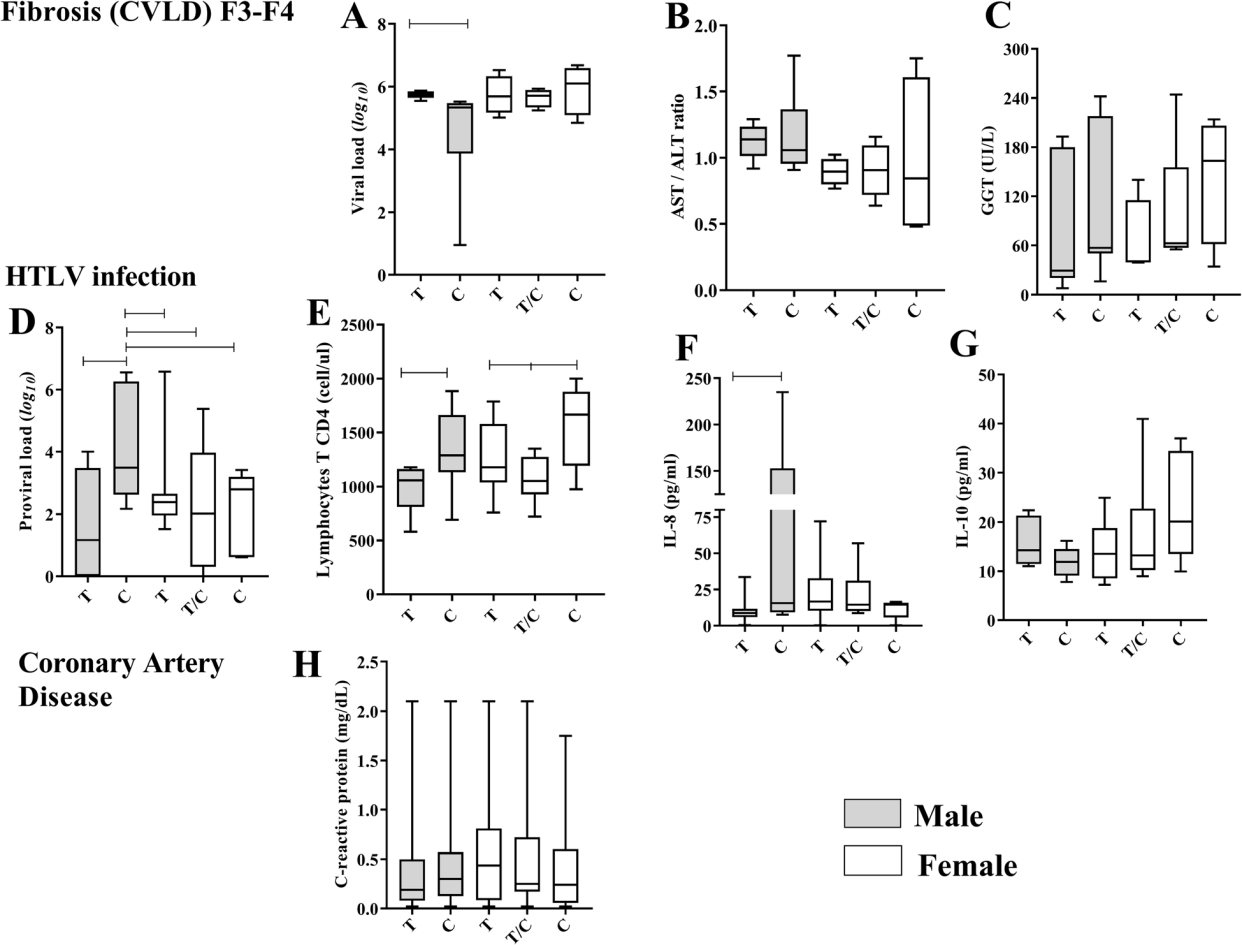
Association of the rs2232365 polymorphism with biomarkers of the pathologies: In men with chronic viral liver diseases and advanced fibrosis, the viral load was higher in patients with the allele T (**a**); the ratio of transaminases (AST/ALT) was higher in men, however, with no differences between alleles (**b**); the level of GGT fluctuated between genotypes/sexes (**c**). In men infected with HTLV-1, the proviral load (**d**), T CD4^+^ lymphocytes (**e**) and the level of IL-8 (**f**) were low in allele T carriers; the level of IL-10 was biased lower in patients with the allele T (**g**), but without statistical significance. (**h**) In CAD patients, CRP levels were statistically similar between genotypes/sexes

In the present study, no significant association between sex and chronic viral liver diseases was observed, although the prevalence of males was higher among those infected. The frequency of polymorphic variants was the same between the sexes when the viral hepatopathy itself was evaluated. (Table 1). In males the allele T was associated with advanced fibrosis, with a relative risk of 4 fold in the bivariate analysis (p:0.05; OR:4.18; CI 95%: 1.13–15.4). In multiple logistic regression the risk of the genetic factor is reduced, however, it still maintains the significance (0.05 ≥ *p* ≥ 0.01; OR:1.11; CI 9%:0.43–2.90) (Table 1). The viral load (VL) was high among males carrying allele T (p: 0.0007) as compared to carriers of the allele C (Fig. 2a), but the transaminase ratio (AST/ALT) was not significant between alleles and sex, although in males this rate is increased (Fig. 2b). The levels of gamma-glutamyl transferase (GGT) in males with allele C were higher than in those with allele T, but without significant differences (Fig. 2c). These associations were not observed in women. Hepatic inflammation was not associated with the gender or genetic profile of those infected (Table 1).

Our data indicate that women are more associated with HTLV-1 than the rs2232365 polymorphism itself (p:0.001; OR: 1.20; CI 95%:0.63–2.29), although the allele T has been associated as a protective factor against infection, especially in males (p:0.04; OR:0.46; CI 95%:0.18–1.18) (Table 1). Again in men, variant T was also associated with low levels of proviral load (p: 0.0396), T CD4^+^ lymphocytes (p: 0.0483) and IL-8 cytokine (p: 0.0549); the elevation of the levels of the cytokine IL-10 was a trend observed, however, with low statistical power (p: 0.1102) (Fig. 2d-g). The T CD8^+^ lymphocytes count was similar between genotypes/sexes (data not shown). Both gender and polymorphism were not associated with an asymptomatic carrier or with more advanced infection profiles (HAM / TSP) (Table 1).

The risk of coronary heart disease was associated only with males, although the significance was lost when analyzing the history of risk infection by *Chlamydia* (Table 1). The frequency of polymorphism was not associated with either the pathological profile or the history of risky infection in both sexes (Table 1). *Chlamydia pneumoniae* was the most frequent species in the study, however, the infection was not associated with the sex and genetic profile of the patients (Table 1). Levels of C-reactive protein (CRP) were low and similar between sexes and alleles (Fig. 2h).

To avoid false positive claims, we infer the value of false positive report probability (FPRP) for all significant associations. All findings remained noteworthy in the prior probability between 0.25 to 0.1; except for CAD (Female vs Male), whose notability is evident from the 0.01 probability (Table 2).

**Table 2:** Calculation of the false-positive report probability (FPRP) for the risk associations of sex and rs2232365 polymorphism to chronic diseases and their clinical and pathological profiles

Associations	OR (IC 95%)	p	Statistical power	Prior probability					
				0.25	0.1	0.01	0.001	0.0001	0.00001
**Sexual associations**									
HTLV-1:									
Female vs Male	1.91 (1.10–3.33)	0.03	0.1971	0.2913	0.5331	0.9113	0.9913	0.9990	0.9999
CAD:									
Female vs Male	1.53 (1.08–2.16)	0.02	0.4552	0.1101	0.2557	0.7558	0.9717	0.9968	0.9997
**Genetic associations**									
HTLV-1:									
(Males) T vs C	0.36 (0.14–0.92)	0.05	0.0990	0.5441	0.7683	0.9676	0.9970	0.9997	1.0000
CVLD (F0-F1):									
(Males) T vs C	4.18 (1.13–15.4)	0.05	0.0617	0.6480	0.8364	0.9787	0.9980	0.9997	0.9999
**Multiple regression associations**									
HTLV-1:									
Sex (Female vs Male)	2.11 (1.18–3.75)	0.02	0.1224	0.2432	0.4717	0.8893	0.9889	0.9986	0.9999
(Males) T vs C	0.43 (0.20–0.95)	0.04	0.1391	0.4885	0.7262	0.9598	0.9962	0.9996	0.9999
CAD:									
Sex (Female vs Male)	1.44 (0.95–2.19)	0.02	0.5757	0.3556	0.6052	0.9324	0.9935	0.9993	0.9999
CVLD (F0-F1):									
(Males) T vs C	1.11 (0.43–2.90)	0.05	0.7306	0.8037	0.9192	0.9903	0.9991	0.9999	0.9999

*CVLD* Chronic Viral Liver Diseases

*CAD* Coronary Artery Disease

## Discussion

There are controversies regarding the genetic changes caused by the rs2232365 polymorphism [6, 9]; however, based on the analysis of gene expression, it is suggested that the polymorphisms alter the expression of *FOXP3*, predisposing the individual to different clinical and pathological outcomes related to the disease in question. From an immunological point of view, there is a tendency for the T allele to favor an anti-inflammatory profile, while the C allele favors a pro-inflammatory profile.

It seems counterintuitive to associate an anti-inflammatory factor with the risk of tissue aggression; however, these findings corroborate previous studies that suggest that variant T favors the persistence of viral infection in patients with fibrosis [10]. With the increase in VL, there is a long-term pro-fibrogenic tendency induced by chronic inflammation and continuous response to healing [11], resulting in the fluctuation of pre-cirrhotic biomarkers [12], such as GGT. The transaminase ratio was stable between genotypes and sexes, indicative of chronic infection, but not relevant for estimating the stage of fibrosis [13]. Recent studies by our group show that in liver fibrosis, viral load, in fact, is more associated with the histological profile than liver integrity enzymes [14].

In the multiple logistic regression, the allele T was also associated with advanced fibrosis; however, the decrease in the statistical probability is indicative of other factors influencing liver histopathology. In recent publications, we discard alcoholism as a behavioral factor associated with liver fibrosis [14].

In HTLV-1 infection, the high prevalence of infected women is an epidemiological fact observed in different populations studied [15, 16], and is related to the effectiveness of sexual transmission from male to female. With the present study, we suggest that not only transmissivity, but also sex-linked immunogenetic factors can influence susceptibility to HTLV-1 infection. The observed associations suggest that intrinsic mechanisms regulate the action of *FOXP3* variants between sexes. Studies show that, in women, epigenetic processes modulate the expression of *FOXP3* and alter the susceptibility to diseases [17]; the normal development of cells carrying mutant genes is a consequence of mixed chimerism [18], which is suggested in the present study due to the marked frequency of heterozygous women (data not shown). In men, changes in *FOXP3* tend to be more relevant due to heredity [19], regulation induced by the Y chromosome [20] and sex hormones [21].

Although there are no studies on the association of the polymorphism with HTLV-1 infection, it is suggested that, in men, the anti-inflammatory tendency induced by the allele T reduces chronic immune hyperactivity, typically seen in the pathogenesis of the infection, reflecting the decrease in the proviral load and the cytokines of cellular immunity [22]. The relationship of the polymorphism with the T CD4^+^ lymphocytes and IL-8 is indicative of the constitution of an atypical pro-inflammatory immune network [23], however, already observed in HTLV-1 infection, mainly in patients with Adult T-cell leukemia (ATL) [24].

In the present study, we did not associate the polymorphism with the clinical and pathological aspects of HTLV-1 infection. However, given the prevalence of asymptomatic patients observed, a cohort study in patients with allele T in this clinical group can clarify whether, in the long term, there will be polarization for a pro-inflammatory profile and changes in the pathogenesis of infection.

Only males were associated with the risk of CAD, however, unrelated to the history of *Chlamydia* infection. The history of *Chlamydia penumonie* prevailed and CRP levels fluctuated, but both were not associated with sex and polymorphism. Recent reports confirm that men are generally more likely to develop CAD than women, with the highest death rate in middle age. Women, on the other hand, are at greater risk of stroke, which usually occurs at older ages [25].

We defend the legitimacy of our data based on the FPRP values obtained that support the proposed findings. All values remained noteworthy between 0.25 to 0.001 of prior probability, which, according to Wacholder and collaborators, for polymorphisms whose functional data are suggestive of a possible association (as shown in the literature for rs2232365) is the ideal probability range [26].

## Conclusion

In conclusion, the results of the present study indicate a relationship between sex and the polymorphism rs2232365 in gene *FOXP*3, in which gene variants seem to be more associated with men and with different immunological roles that vary between chronic viral infections.

## Methods

A total of 323 females and 373 males diagnosed with chronic viral liver disease (CVLD), human T-cell leukemia virus type 1 (HTLV-1) infection or coronary artery disease (CAD) and healthy groups of blood donors, all from regional reference centers in the state of Pará, Brazil (Tropical Medicine Nucleus of the Federal University of Pará, Foundation of Hemotherapy and Hematology Center of Pará (HEMOPA) and Institute of Health Sciences of the Federal University of Pará), were evaluated. Participants were informed about the objectives of the study and, after agreeing to participate, signed a consent form. The study was approved by the ethics committees of the participating entities (CAAE: 73782017.8.0000.0018; CAAE: 0011.0.324.000–09 and CAAE: 31223214.2.0000.0018). All patients were not undergoing treatment.

The inclusion/exclusion criteria and the biological data for patients CVLD were established in our previous study [10, 14].

Patients HTLV-1^+^ were classified as asymptomatic or with paraparesis (HTLV-1-associated myelopathy/tropical spastic paraparesis - HAM/TSP) according to pre-established criteria [27]. The quantification of the proviral load was based on previously described protocols [27, 28]. Quantification of lymphocytes TCD4^+^ and CD8^+^ was performed by immunophenotyping in flow cytometry (FACSCountTM Reagents - TriTEST™/TruCount, BD Biosciences, San Jose, CA, EUA). Pro-inflammatory (TNF-α, TNF-β, IFN-γ, IL6 and IL8) and anti-inflammatory (IL-10) cytokine levels were determined by immunoenzymatic assays (Human ELISAReady-SET-Go, EBioscience, Inc., San Diego, CA, USA).

Patients with coronary disease had arterial obstruction presenting with severe arterial obstruction with or without ischemia (infarction) and another group of patients with cardiac valvulopathy, presenting with volume overload and cardiac pressure. The history of *Chlamydia* infection in these patients was obtained by immunoenzymatic assays (NovaTec Immundiagnostica GmbH, Germany); and C-reactive protein (CRP) levels were measured by immunoturbidimetry (DiaSys, Waterbury, CT, USA).

Genotyping of the polymorphism (rs2232365) was performed by real-time PCR (C_15942641_10 - Applied Biosystems, Foster City, CA, USA) with established temperature and cycling protocols [10]. To explore the biological potential of the polymorphism, we evaluated the gene expression of genotypes based on the public database of the GTex portal (https://www.gtexportal.org/home/snp/rs2232365).

The estimation of the Hardy-Weinberg equilibrium was performed using the chi-square test, in which only the genotype data of the women (diploids) were used, because the *FOXP3* gene is located on the human sex chromosome (X on XY model).

The chi-square test and G test were applied to assess the association of sex and the frequency of polymorphism with the clinical and pathological aspects of chronic diseases, following the recommendations of each test; Fisher’s Exact test was applied with the same intention, however, for comparisons arranged in 2 × 2 contingency tables. Followed by the calculation of *Odds ratio* (OR) to determine the advantage (or disadvantage) of the factors with significant associations. The calculation of multiple logistic regression was proposed to assess the degree of dependence of clinical and pathological variables on sex and the frequency of polymorphism. For CVLD and CAD, we assume the male gender and the frequency of the T allele as “1” (success) and the female gender and the frequency of the C allele as “0” (failure). For HTLV-1 infection, we assume the female sex and the frequency of the T allele as “1”; the male gender and the frequency of the C allele as “0”.

The Kruskal-Wallis test was used to calculate the association of the polymorphism with the quantitative biomarkers of the studied pathologies. The Mann-Whitney test was used as a confirmatory method, this time, comparing two independent samples.

To calculate the probability of false positives for significant associations, we predefine an FPRP limit value of 0.5. The *Odds ratio* was 1.5 for the calculation of statistical power. Associations that remained noteworthy between the ranges of 0.25 to 0.01 of prior probability were considered true positive, according to Wacholder’s recommendations [26].

Statistical analysis was performed using BioEstat 5.0 [29] and GraphPad Prism 6.1, with a significance level of 95% (*p* ≤ 0.05). FPRP was calculated using R 3.4.2 software [30].

## Data Availability

The original data sets generated and analyzed during this study are made available by the corresponding author upon reasonable request. Gene expression data was collected from the public database of the Gtext portal (https://www.gtexportal.org/home/snp/rs2232365).

## Abbreviations

rs2232365: Polymorphism of the substitution of thymine base for a cytosine base in the promoter region (− 924) of the FOXP3 gene
ALT: Alanine transaminase
AST: Aspartate transaminase
CAD: Coronary Artery Disease
CVLD: Chronic Viral Liver Diseases
CRP: C-reactive protein
FOXP3: Forkhead box protein 3
FPRP: False positive report probability
GGT: Gamma-glutamyl transferase
HAM/TSP: HTLV-1-associated myelopathy/tropical spastic paraparesis
HTLV-1: Human T-cell leukemia virus type 1
Treg: Regulatory T-cell
VL: Viral load
